# Genetic susceptibility to high myopia in Han Chinese population

**DOI:** 10.1515/biol-2022-0055

**Published:** 2022-05-17

**Authors:** Yufei Li, Yong Zhang, Ping Zhang, Lei Gao, Qingyue Ma, Jin Li, Shengxia Wang, Bing Liu, Xinye Wang, Chao Meng

**Affiliations:** College of Life Sciences, Shandong First Medical University & Shandong Academy of Medical Sciences, Tai’an, 271016, China; Continuous Education College, Shandong First Medical University & Shandong Academy of Medical Sciences, Tai’an, 271016, China; Infirmary, Shandong First Medical University & Shandong Academy of Medical Sciences, Tai’an, 271016, China; Graduate School, Shandong First Medical University & Shandong Academy of Medical Sciences, Tai’an, 271016, China; College of Pharmacy, Shandong University, Ji’nan, 250100, China; Ophthalmology Department, Tai’an City Central Hospital, Tai’an, 271000, China; College of Basic Medicine, Shandong First Medical University & Shandong Academy of Medical Sciences, Tai’an, 271016, China

**Keywords:** high myopia, single nucleotide polymorphism, *IGF-1*, *MMP9*, association study

## Abstract

High myopia is a common ocular genetic disease in the world. The study sought to investigate the effect of the Insulin-like growth factor-1 (IGF-1) and Matrix metalloproteinase-9 (MMP-9) genes polymorphisms on high myopia in a Han population of China. This study recruited 216 unrelated Han Chinese subjects, including 103 cases with high myopia and 113 controls. Four tagging single nucleotide polymorphisms (SNPs) of *IGF-1* and *MMP-9* genes were genotyped using the Sequenom MassARRAY method. The chi-square test showed that the family history was significantly correlated with myopia. The SNP genotypes were all in Hardy–Weinberg equilibrium (*P* > 0.05). Among the four SNPs, there were statistically significant differences in the genotype and allele frequencies of *rs2236416* between the groups (*P* = 0.024). The significant associations of *rs2236416* between cases and controls also appeared after Bonferroni multiple correction (*P* = 0.024). Then, there were significant differences in the genotypes dominant model and codominant model of *rs2236416* between groups (*P* = 0.007 and *P* = 0.004, respectively). *rs5742632* showed a significant difference between the cases and the controls in the recessive model (*P* = 0.037). Our findings indicated that *rs2236416* of *MMP-9* was associated with myopia in the population. The result suggested *MMP-9* gene locus may play a role in myopia.

## 1 Introduction

High myopia is a common genetic eye disease characterized by axis oculi elongation and pathological changes. The incidence of myopia is increasing and has become one of the major public health problems [1]. Notably, the incidence of high myopia in Chinese high school students has reached 10–20%, while among college and graduate students, the rate has reached 18 and 23%, respectively [2,3]. Myopia causes serious complications, including retinal detachment, macular degeneration, and vision loss [4]. There is no effective way to treat and prevent high myopia now. Family and twin studies have shown a high heritability of high myopia [5,6,7]. Candidate genes, including collagen genes, growth factor genes, and transcription factor genes, have been found to be physically linked with genetic markers of myopia [8]. Insulin-like growth factor-1 (*IGF-1*) is a pluripotent cytokine exerting its effects in virtually all cell types and plays a vital role in the nervous system [9]. *IGF-1* gene also regulates the growth of eyeballs. Matrix metalloproteinase-9 (*MMP-9*) could remodel and degrade extracellular matrix (ECM). The genes may play an important role in the occurrence and development of high myopia [10,11]. It is shown that the potential association between *IGF-1* polymorphisms and myopia has been investigated in a recent study [12]. However, the association studies of *IGF-1* and *MMP-9* genes were performed in a different population, and more single nucleotide polymorphisms (SNPs) on the way [13,14]. The signal for establishing the causes of myopia serves as a tool for the development of research [15].

Here we verify the hypothesis that these polymorphisms were the genetic factors that may influence myopia. Therefore, the present study will explore the association of polymorphisms with myopia and possible pathogenic factors in a Han population. A total of four SNPs in *IGF-1* and *MMP-9* were selected, including *rs5742632*, *rs10860860*, *rs17576,* and *rs2236416*.

## 2 Materials and methods

### 2.1 Participants

One hundred and three cases with high myopia were recruited. The subjects who met the following criteria were selected for the current study: (1) age between 17 and 23 years, (2) spherical equivalent (SE) in the eye of more than −6.00 diopter (D), (3) axial length more than or equal to 26 mm, and (4) without ocular disease and systemic diseases. One hundred and thirteen cases of normal control group had a SE less than −0.5 D, axial length less than 26 mm, and without systemic diseases. Cases and controls were both Han Population of Shandong Province.


**Informed consent:** Informed consent has been obtained from all the individuals included in this study.
**Ethical approval:** The research related to human use has been complied with all the relevant national regulations, institutional policies and in accordance with the tenets of the Helsinki Declaration, and has been approved by the Ethics Committee of Shandong First Medical University.

### 2.2 DNA extraction

Peripheral venous blood (5 mL) of the subjects was collected, placed in EDTA blood vessels, numbered, and stored at –80°. Genomic DNA was extracted using TIANamp Genomic DNA Kit (Tiangen Biotech (Beijing) Co., Ltd), according to the instructions. The quality of the DNA was identified by agarose gel electrophoresis.

### 2.3 Selection and genotyping of SNPs

Based on HapMap data for Han Chinese, the taggers used the following criteria for SNP selection: pairwise tagging algorithm, *r* ^2^ > 0.8, and minor allele frequency (MAF) >0.05. The selected candidate sites, *rs5742632*, *rs10860860*, *rs17576*, and *rs2236416*, were available in GenBank.

Genotyping was performed by using the Sequenom MassARRAY platform. The primers were designed by Assay Design 3.1 software.

### 2.4 Statistical analysis

Genotype and allelic frequencies were calculated, and the data were tested for Hardy–Weinberg (HWE) equilibrium using SPSS 20.0 software. The differences of genotype and allele frequencies were compared among the groups by chi-square test. Logistics regression analysis was used to calculate the genotype and allele ratio (odds ratio) and 95% confidence interval ( CI). Bonferroni’s correction was applied for multiple comparisons. *P* < 0.05 was defined as statistical significance.

## 3 Results

### 3.1 Study participants

The study included 216 unrelated Chinese Han population subjects, including 103 cases (64 female and 39 male, age 17–20) with high myopia and 113 controls (67 female and 46 male, age 17–20). There was no significant difference in age and gender between the cases and controls (*P* = 0.212 and *P* = 0.671, respectively). The proportion of cases with family history was 55.4%. The prevalence of myopia among students whose parents were not myopic was 44.66%. The data showed an association between family history and the presence of myopia (*P* < 0 .001).

### 3.2 SNP analysis and genetic association study

The selected SNPs were successfully genotyped within HWE in both the groups (*P* > 0.05, Table 1). The information on the SNPs is shown in Table 1. There were significant differences in genotype distributions of *rs2236416* between the two groups after Bonferroni correction (*P* = 0.024) (Table 1). There were significant differences between the genotypes dominant and codominant models of *rs2236416* between groups (*P* = 0.007 and *P* = 0.004, respectively). *rs5742632* showed a significant difference between the cases and the controls in the recessive model (*P* = 0.037) (Table 2).

**Table 1:** Genotype distributions for the SNPs in groups

SNP	Location	Alleles	Minor alleles	Genotype	Case	Control	*P*-value	HWE-P
*rs5742632*	102462696	A	G	GG/GA/AA	14/56/29	29/51/33	0.090	0.116
*rs10860860*	102387055	A	T	AA/AT/TT	68/27/6	73/34/5	0.777	0.153
*rs17576*	46011586	G	A	AA/AG/GG	5/48/50	7/54/52	0.877	0.123
*rs2236416*	46011936	A	G	AA/GA/GG	88/14/1	79/31/3	0.024	0.603

HWE-p, the *P* value of Hardy–Weinberg equilibrium.

**Table 2:** Genotype distributions for the 4 SNPs in high myopia and control groups

				Codominant		Dominant		Recessive	
SNP	Genotype	Case	Control	OR (95%CI)	*P*	OR (95%CI)	*P*	OR (95%CI)	*P*
*rs5742632*	GG	14	29	1.140 (0.642–2.025)	0.654	0.996 (0.550–1.802)	0.989	2.096 (1.035–4.244)	0.037
	GA	56	51						
	AA	29	33						
*rs10860860*	AA	68	73	1.051 (0.611–1.809)	0.858	1.101 (0.623–1.945)	0.741	0.740 (0219–2.502)	0.627
	AT	27	34						
	TT	6	5						
*rs17576*	AA	5	7	1.127 (0.668–1.902)	0.653	1.107 (0.648–1.889)	0.710	1.294 (0.398–4.212)	0.668
	AG	48	54						
	GG	50	52						
*rs2236416*	AA	88	79	2.576 (1.331–4.986)	0.004	2.525 (1.280–4.980)	0.007	2.782 (0.285–27.174)	0.359
	AG	14	31						
	GG	1	3						

OR, odds ratio; CI, confidence interval.

The mean age of onset with the G allele of *rs2236416* SNP (G/G or G/A genotype; 11.00 ± 0.000 years or 11.29 ± 1.773 years) was lower than those without (A/A genotype; 11.42 ± 1.910 years), which did not reach a statistical significance (*P* = 0.104). There were no significant differences between the age of onset and the genotype distribution of *rs5742632, rs1086086,0,* and *rs17576* (*P* = 0.902, *P* = 0.342, and *P* = 0.870, respectively) (Table 3).

**Table 3:** Association between genotypes of 4 SNPs and age of onset in case group

SNP	Genotype	Age of onset	SD	*F* value	*P* value
*rs5742632*	AA/GA/GG	11.48/11.29/11.36	1.844/1.826/2.240	0.104	0.902
*rs10860860*	AA/AT/TT	11.51/11.00/12.00	1.706/2.201/1.549	1.085	0.342
*rs17576*	AA/AG/GG	11.20/11.32/11.50	1.924/1.810/1.963	0.140	0.870
*rs2236416*	AA/GA/GG	11.42/11.29/11.00	1.910/1.773/0.000	0.053	0.948

SD standard deviation; *F* value for the joint hypotheses test.

## 4 Discussion

The study explored the association between the SNPs (*rs5742632*, *rs10860860*, *rs17576*, and *rs2236416*) of *IGF-1* and *MMP-9* and high myopia in a Han population. Consistent with expectations, we found a significant association between *rs2236416* and high myopia. These results are, to some extent, in line with the previous research [13,15].

*IGF-1* gene is located in the MYP3 locus on chromosome 12q23.2, which is similar to insulin in structure and takes part in many physiological processes, including aging, apoptosis, development, cellular growth metabolism, and protein translation [16]. It is a single-chain peptide composed of 70 amino acids which play an important role in human growth and development [17]. IGF-1 has been listed as a potential myopia-related gene in animal experiments and in-body research and is considered to play an important role in controlling eyeball growth. The associations between two SNPs of *IGF-1* (*rs10860860* and *rs2946834*) and high myopia (*P* < 0.05) have been indicated to be significant in a Polish population [18]. *rs2162679* in *IGF-1* was associated with myopia in a young Chinese population [12]. These results do not agree with our findings. The phenomenon may be due to different geographical ethnic groups. Our result was confirmed by additional analyses confirmed in Japanese patients [19]. Miyake reported *rs6214* and *rs5742632* were not associated with high myopia from the different statistical analysis methods [19]. Furthermore, Zidan demonstrated that *rs6214* was associated with high myopia in an Egyptian population [20]. The phenotype of high myopia-causing genes may change as a result of ethnic differences. The varied genetic background of patients may also lead to different results in the association studies.

*MMP-9* gene, located on chromosome 20q11.2-q13.1, is an endopeptidase that is considered as an important factor in the development of the axial length of the eye. The established study suggested that *MMP-9* could remodel and degrade the extracellular matrix (ECM) [21]. It has an important effect on the growth and development process of the eye by cleaving denatured collagen and type IV collagen in the basement membrane [22]. The association studies became focused on the effects of *MMP-9* on glaucoma [23]. According to clinical statistics, patients with myopia have higher intraocular pressure and are more susceptible to open-angle glaucoma. The debate about the role of myopia in the progress of glaucoma is still continuing. Schache found no association between *rs17156* and high myopia in an Australian population [14]. However, our findings suggested that *rs2236416* was significantly associated with high myopia in the Han Chinese population. This conclusion was confirmed in another report [24]. rs2236416 is an intron variant. Although the precise molecular mechanism observations are unclear, a possible explanation is that this variant of the MMP-9 gene may lead to the modified expression of this gene in high myopia.

Family history has an important effect on the occurrence of myopia, especially when both parents suffer from this disease [25]. Pacella investigated that the probability of children with two myopia parents falling ill to the disease was 6.42 times higher than children with one or both healthy parents [26]. Similar to the result, this study shows that cases with two or one myopia parents are more likely to develop myopia (*P* < 0.001) than without myopia parents.

The association between genes and the age of onset of high myopia had been found in previous research [27,28]. In the present study, question regarding the relationship between polymorphisms and the age of onset of high myopia also appeared. An unexpected answer is that no significant association was found. Large sample size is needed to give a certain level of confidence in further research. Further replication studies are needed to validate our findings.

## 5 Conclusion

We found the significant effect of genetic factors on high myopia and identified an association between *rs2236416* and high myopia in some Han Chinese populations. The study focused on some new loci in a specific population. Interestingly, the associations between *rs5742632* and high myopia have been indicated to be significant in the genotypes recessive model. This conclusion requires further research. The polymorphisms were significantly correlated to the occurrence risk of high myopia. The association of the polymorphisms with family history and the age of onset of high myopia were investigated. Larger sample size and more SNPs are needed to explore the mechanism of myopia.

## References

[1] Pan CW, Ramamurthy D, Saw SM. Worldwide prevalence and risk factors for myopia. Ophthalmic Physiol Opt. 2012;32(1):3–16. 10.1111/j.1475-1313.2011.00884.x 22150586

[2] Wu LJ, You QS, Duan JL, Luo YX, Liu LJ, Li X, et al. Prevalence and associated factors of myopia in high-school students in Beijing. PLoS One. 2015;10(3):e0120764. 10.1371/journal.pone.0120764 PMC4372519 25803875

[3] Sun J, Zhou J, Zhao P, Lian J, Zhu H, Zhou Y, et al. High prevalence of myopia and high myopia in 5060 Chinese university students in Shanghai. Invest Ophthalmol Vis Sci. 2012;53(12):7504–9. 10.1167/iovs.11-8343 23060137

[4] Li L, Cui YJ, Zou Y, Yang L, Yin X, Li B, et al. Genetic association study of SOX2 gene polymorphisms with high myopia in a Chinese population. Eur J Ophthalmol. 2021;31(2):734–9. 10.1177/1120672120904666 32037877

[5] Tang WC, Yap MK, Yip SP. A review of current approaches to identifying human genes involved in myopia. Clin Exp Optom. 2008;91(1):4–22. 10.1111/j.1444-0938.2007.00181.x 18045248

[6] Hornbeak DM, Young TL. Myopia genetics: a review of current research and emerging trends. Curr Opin Ophthalmol. 2009;20(5):356–62. 10.1097/ICU.0b013e32832f8040 PMC3736551 19587595

[7] Dirani M, Shekar SN, Baird PN. Adult-onset myopia: the genes in Myopia (GEM) twin study. Invest Ophth Vis Sci. 2008;49(8):3324–7. 10.1167/iovs.07-1498 18408192

[8] Zhang X, Zhou X, Qu X. Association between COL1A1 polymorphisms and high myopia: a meta-analysis. Int J Clin Exp Med. 2015;8(4):5862–8. PMC4483928 26131177

[9] Liu C, Liu S, Wang S, Sun Y, Lu X, Li H, et al. IGF-1 via PI3K/Akt/S6K signaling pathway protects DRG neurons with high glucose-induced toxicity. Open Life Sci. 2019;14(1):502–14. 10.1515/biol-2019-0056 PMC7874800 33817186

[10] González Blanco F, Sanz Ferńandez JC, Muńoz Sanz MA. Axial length, corneal radius, and age of myopia onset. Optom Vis Sci. 2008;85(2):89–96. 10.1097/OPX.0b013e3181622602 18296925

[11] Chen X, Chen Y, Wiggs JL, Pasquale LR, Sun X, Fan BJ. Association of MATRIX metalloproteinase-9 (MMP9) variants with primary angle closure and primary angle closure glaucoma. PLoS One. 2016;11(6):e0157093. 10.1371/journal.pone.0157093 PMC4896618 27272641

[12] Cheng T, Wang J, Xiong S, Zhang B, Li Q, Xu X, et al. Association of IGF1 single-nucleotide polymorphisms with myopia in Chinese children. PeerJ. 2020;8:e8436. 10.7717/peerj.8436 PMC6991122 32025377

[13] Yoshida M, Meguro A, Yoshino A, Nomura N, Okada E, Mizuki N. Association study of IGF1 polymorphisms with susceptibility to high myopia in a Japanese population. Clin Ophthalmol. 2013;7:2057–62. 10.2147/OPTH.S52726 PMC3804590 24204106

[14] Schache M, Baird PN. Assessment of the association of matrix metalloproteinases with myopia, refractive error and ocular biometric measures in an Australian cohort. PLoS One. 2012;7(10):e47181. 10.1371/journal.pone.0047181 PMC3471969 23077567

[15] Dashdamirova GS, Alieva KA. Medical-genetic study of the population of the Lenkoran district of the Azerbaijani Republic. Cytol Genet. 2009;43(4):262–66. 19938647

[16] Bartke A. The somatotropic axis and aging: mechanisms and persistent questions about practical implications. Exp Gerontol. 2009;44(6–7):372–4. 10.1016/j.exger.2009.04.001 PMC4207209 19371777

[17] Savage MO. Insulin-like growth factors, nutrition and growth. World Rev Nutr Diet. 2013;106:52–9. 10.1159/000342577 23428681

[18] Metlapally R, Ki CS, Li YJ, Tran-Viet KN, Abbott D, Malecaze F, et al. Genetic association of insulin-like growth factor-1 polymorphisms with high-grade myopia in an international family cohort. Invest Ophthalmol Vis Sci. 2010;51(9):4476–9. 10.1167/iovs.09-4912 PMC2941166 20435602

[19] Miyake M, Yamashiro K, Nakanishi H, Nakata I, Akagi-Kurashige Y, Tsujikawa A, et al. Insulin-like growth factor 1 is not associated with high myopia in a large Japanese cohort. Mol Vis. 2013;19:1074–81. PMC3668686 23734076

[20] Zidan HE, Rezk NA, Fouda SM, Mattout HK. Association of insulin-like growth factor-1 gene polymorphisms with different types of myopia in egyptian patients. Genet Test Mol Biomarkers. 2016;20(6):291–6. 10.1089/gtmb.2015.0280 27167306

[21] Nagase H, Woessner JF Jr. Matrix metalloproteinases. J Biol Chem. 1999;274(31):21491–4. 10.1074/jbc.274.31.21491 10419448

[22] Mathalone N, Marmor S, Rahat MA, Lahat N, Oron Y, Geyer O. MMP expression in leaking filtering blebs and tears after glaucoma filtering surgery. Graefes Arch Clin Exp Ophthalmol. 2011;249(7):1047–55. 10.1007/s00417-011-1658-0 21452038

[23] Park HL, Hong KE, Park CK. Impact of age and myopia on the rate of visual field progression in glaucoma patients. Medicine. 2016;95(21):e3500. 10.1097/MD.0000000000003500 PMC4902340 27227916

[24] Hall NF, Gale CR, Ye S, Martyn CN. Myopia and polymorphisms in genes for matrix metalloproteinases. Invest Ophthalmol Vis Sci. 2009;50(6):2632–6. 10.1167/iovs.08-2427 19279308

[25] Morgan IG, Ohno-Matsui K, Saw SM. Myopia. Lancet. 2012;379(9827):1739–1748. 10.1016/S0140-6736(12)60272-4 22559900

[26] Pacella R, McLellan J, Grice K, Del Bono EA, Wiggs JL, Gwiazda JE. Role of genetic factors in the etiology of juvenile-onset myopia based on a longitudinal study of refractive error. Optom Vis Sci. 1999;76(6):381–6. 10.1097/00006324-199906000-00017 10416932

[27] Annamaneni S, Bindu CH, Reddy KP, Vishnupriya S. Association of vitamin D receptor gene start codon (Fok1) polymorphism with high myopia. Oman J Ophthalmol. 2011;4(2):57–62. 10.4103/0974-620X.83654 PMC3160070 21897619

[28] Verkicharla PK, Kammari P, Das AV. Myopia progression varies with age and severity of myopia. PLoS One. 2020;15(11):e0241759. 10.1371/journal.pone.0241759 PMC7678965 33216753

